# Role of pepper bZIP transcription factor CaADBZ1 in abscisic acid signalling and drought stress response

**DOI:** 10.1111/ppl.70159

**Published:** 2025-03-19

**Authors:** Jihye Choi, Chae Woo Lim, Sung Chul Lee

**Affiliations:** ^1^ Department of Life Science (BK21 program) Chung‐Ang University Seoul Korea

## Abstract

In plants, basic‐region/leucine‐zipper (bZIP) transcription factors are key regulators of stress responses mediated by various phytohormone signalling pathways. However, the roles of bZIP transcription factors in pepper, particularly those associated with ABA signalling and drought stress, remain poorly understood. In this study, we isolated the *CaADBZ1* (*Capsicum annuum* ABA and Dehydration‐Induced bZIP transcription factor 1) gene, a member of the group A family, and analysed its functions in response to dehydration stress and ABA signalling. The expression of *CaADBZ1* was specifically induced by dehydration and exogenous ABA treatment, not salinity and osmotic stress. CaADBZ1 was found to have transactivation activity in yeast cells, which was dependent on the N‐terminal of CaADBZ1 (amino acids 1–112), harbouring a highly conserved C1 domain. Notably, a dual‐luciferase reporter assay revealed that CaADBZ1 modulated the expression of *CaOSR1*, a dehydration stress‐responsive gene in pepper plants. Functional studies in both pepper and Arabidopsis plants revealed that the modulation of *CaADBZ1* expression level affected dehydration stress resistance in pepper and Arabidopsis plants. *CaADBZ1*‐silenced pepper *Arabidopsis* plants showed dehydration stress‐sensitive phenotypes characterized by higher transpiration rates and reduced expression of dehydration‐responsive genes compared to control plants. Conversely, overexpression of the *CaADBZ1* gene in Arabidopsis plants enhanced dehydration stress resistance. Moreover, *CaADBZ1*‐overexpressing *Arabidopsis* transgenic plants showed increased ABA sensitivity during the seedling stage. Collectively, our findings suggest that CaADBZ1 plays a crucial role in enhancing dehydration stress tolerance in plants by positively regulating ABA sensitivity and dehydration‐responsive gene expression.

## 1 INTRODUCTION

Due to ongoing global climate abnormalities and extreme weather events, plant growth and development are being atypically affected by a range of abiotic stresses, thereby ultimately influencing their survival. Among these abiotic stresses, water deficit‐induced dehydration represents one of the most critical challenges to agriculture, as it leads to substantial reductions in crop yield (Dietz et al., 2021; Hendrawan et al., 2023). To ensure survival when exposed to dehydration stress, plants have evolved a range of physiological and morphological defence mechanisms, including the closure of stomata and the expression of stress‐related genes (Brodribb & Holbrook, 2003; Yamaguchi‐Shinozaki & Shinozaki, 2006). One of the most prominent defence mechanisms is stomatal closure, which minimizes water loss and is primarily regulated by the plant hormone abscisic acid (ABA). (Bharath et al., 2021; Davies et al., 2002).

The core components of the ABA signalling pathway comprise the PYR/PYL/RCAR ABA receptor, clade A type‐2C protein phosphatase (PP2Cs), and non‐fermenting 1‐related subfamily 2 protein kinases (SnRK2s) (Ma et al., 2009; Park et al., 2009; Umezawa et al., 2009). Under normal growth conditions, PP2Cs bind to SnRK2s and dephosphorylate them, thereby inhibiting their kinase activity (Hirayama & Umezawa, 2010; Umezawa et al., 2010). However, in response to dehydration stress, plants synthesize and accumulate ABA (Tuteja, 2007; Vishwakarma et al., 2017). The perception of ABA by its receptors promotes the inhibition of clade A PP2Cs, thereby releasing the suppression of SnRK2s (Cutler et al., 2010; Yoshida et al., 2006). This activation of SnRK2s triggers the phosphorylation of downstream targets, including ABA‐responsive transcription factors such as ABRE‐BINDING PROTEINS (AREBs) and ABRE‐BINDING FACTORS (ABFs), which in turn regulate the expression of stress‐responsive genes (Soma et al., 2021; Takahashi et al., 2018). AREBs and ABFs are known to induce the expression of late embryogenesis abundant (LEA) genes, including *RESPONSIVE TO DESSCICTION 29B* (*RD29B*),  *RESPONSIVE TO ABA 18* (*RAB18*) (Fujita et al., 2005; Yoshida et al., 2010). Additionally, these transcription factors have been found to regulate stomatal closure by targeting genes, such as *ACTIN‐DEPOLYMERIZING FACTOR 5* (*ADF5*), in vegetative tissues (Collin et al., 2021; Qian et al., 2019).

Transcription factors typically comprise four main elements: a DNA binding region, an oligomerization site, a transcription regulation domain, and a nuclear localization signal (Liu et al., 1999). Based on these structural features, plant transcription factors are broadly categorized into 13 groups. Among these, the basic leucine zipper (bZIP) transcription factors are characterized by a highly conserved bZIP domain, which comprises two structural elements positioned on a continuous alpha‐helix. (Hurst, 1994). One element is the basic region containing approximately 18 amino acid residues adjacent to a conserved N‐x7‐R/K‐x9 motif that are crucial for nuclear localization and DNA binding, respectively. The second element, a leucine zipper, forms an amphipathic helix with heptad repeats of leucine or other large hydrophobic amino acids spaced nine residues from the C‐terminus (Jakoby et al., 2002; Nijhawan et al., 2008). Previous studies have revealed that the bZIP transcription factor plays a key role in various developmental processes such as seed germination, maturation, and flower and vascular development (Jakoby et al., 2002; Shiota et al., 2008; Sornaraj et al., 2016; Toh et al., 2012; Yin et al., 1997). Additionally, bZIP transcription factors are implicated in the regulation of ABA signalling and plant stress responses, such as drought and pathogen infection (Alves et al., 2013; Hsieh et al., 2010; Liao et al., 2008; Uno et al., 2000). For example, ABA Insensitive 5 (ABI5), a bZIP transcription factor, positively modulates ABA‐dependent biological processes such as seed dormancy and stomatal closure (Guo et al., 2022; Lee et al., 2012; Lopez‐Molina et al., 2001; Reeves et al., 2011; Yu et al., 2015). ABI5 recognizes and binds to G‐boxes, also known as ABA‐responsive elements (ABREs), within the promoters of ABA‐responsive genes, thereby enhancing their transcriptional expression (Carles et al., 2002; Choi et al., 2000).

In this study, we isolated a group A pepper bZIP transcription factor, CaADBZ1 (*Capsicum annuum* ABA‐ and Drought Induced bZIP transcription factor 1), whose transcript levels are highly induced by exogenous ABA treatment and dehydration stress. Functional assays demonstrated that CaADBZ1 has transcription activity in plants and promotes the expression of the *CaOSR1* gene, a homolog of Arabidopsis *RD29B*, which is a well‐known marker gene for ABA‐ and drought responses. Phenotypic analyses showed that *CaADBZ1*‐silenced pepper plants were found to be characterized by reduced ABA sensitivity and impaired dehydration tolerance, whereas *CaADBZ1*‐overexpressing (OE) Arabidopsis plants showed increased ABA sensitivity at both seedling and adult stages and enhanced dehydration resistance. Furthermore, CaADBZ1 positively regulated the transcription of dehydration‐responsive genes. Collectively, our findings suggest that CaADBZ1 plays a positive role in ABA signalling and dehydration response.

## 2 MATERIALS AND METHODS

### 2.1 Plant materials

As experimental plants, pepper (*Capsicum annuum* cv. Nockwang), *Arabidopsis thaliana* (Columbia‐0 ecotype), and tobacco (*Nicotiana benthamiana*). Pepper seeds were germinated in a growth chamber at 28°C under dark conditions for 5 days, and *Arabidopsis* seeds were initially vernalized at 4°C for 2 days and thereafter germinated on MS medium, which included 1% sucrose and 0.8% Micro agar. Germinated seeds were planted in a pot comprising a steam‐sterilized compost soil mix (peat moss, perlite, and vermiculite, 5:3:2, v/v/v), sand, and loam soil (1:1:1, v/v/v). Tobacco seeds were sown in the same soil mixture. All the plants were grown in a growth room at 24°C with 60% relative moisture under white fluorescent light (130 μmol photons m^−2^ s^−1^).

### 2.2 Subcellular localization analysis

The full‐length coding region of CaADBZ1, which omitted the stop codon, was cloned into the GFP‐fused binary vector p326GFP. The GV3101 strain of *Agrobacterium tumefaciens* contains a gene construct which was infiltrated in *N. benthamiana* with p19 strain in a 1:1 ratio (OD_600_ = 0.5). Two days post‐infiltration, the GFP signals were visualized using an LSM700 confocal microscope (Carl Zeiss), and the localization of the gene was determined using LSM Image Browser software.

### 2.3 Virus‐induced silencing system

A tobacco rattle virus‐induced gene silencing (VIGS) system was used to generate *CaADBZ1*‐silenced pepper plants, as previously outlined (Lim et al., 2021). Briefly, a 300‐bp fragment corresponding to the target sequence of the CaADBZ1‐specific target region was designed using the SGN VIGS tool (http://vigs.solgenomics.net) and subsequently inserted these fragments into a pTRV2 vector. *A. tumefaciens* carrying either pTRV1 and pTRV2:00 as negative control vectors or pTRV2:CaADBZ1 were co‐infiltrated into the cotyledons of pepper plants using a needleless syringe (OD_600_ = 0.2).

### 2.4 Generation of transgenic mutants overexpressing full‐length CaADBZ1

The coding sequences of CaADBZ1 were cloned into a pCR8/GW/TOPO vector (Invitrogen). Using the LR reaction, a *Pro35SCaADBZ1* construct was generated and subsequently a 35S*CaADBZ1* construct. This construct was incorporated then into *A. tumefaciens* strain GV3101, which was subsequently used for the transformation of *Arabidopsis* plants via the floral dip method. To identify transgenic plants, seeds of the transformed plants were grown on Murashige and Skoog (MS) medium containing 25 μg mL^−1^ phosphinothricin.

### 2.5 RNA isolation and real‐time transcription polymerase chain reaction (qRT‐PCR)

Total RNA was extracted from pepper leaves and *A. thaliana* rosette leaves using TRIzol Reagent. To analyze the expression patterns of the CaADBZ1 gene in environmental stimuli, pepper plants at the six‐leaf stage were subjected to dehydration, ABA, saline (NaCl), and mannitol treatments, as previously described (Bae et al., 2023; Lim et al., 2021). Pepper plants are carefully uprooted and subjected to drying, or irrigated with saline (200 mM) or mannitol (600 mM) solutions, or sprayed with 100 μM ABA. Following treatment, the first and second leaves of the plants were harvested at designated time points (0, 2, 6, 12, and 24 h). Quantitation of total RNA was performed using Synergy HTX Multi‐Mode Microplate Reader. cDNA was synthesized using an iScriptTM cDNA synthesis kit (Bio‐Rad) with 1 μg aliquot of the RNA. The resulting cDNA served as a template for qRT‐PCR analysis, following previously outlined procedures (Lim et al., 2021). *Arabidopsis* Actin8 (*AtACT8*) and pepper PP2A (*CaPP2A*) were used to normalize qRT‐PCR data. For qRT‐PCR, the synthesized cDNA was amplified utilizing iQ SYBR Green Supermix and specific primers (Table S1) in a CFX96 Touch Real‐Time PCR detection system (Bio‐Rad).

### 2.6 Plant phenotypic analyses

For seed germination and seedling growth analyses, 36 sterilized seeds of *CaADBZ1*‐OE were plated on an MS agar medium containing different concentrations of ABA. The seeds were initially vernalized for 2 days at 4°C, and subsequent germination rates were recorded in the number of seeds with visible radicle presence over 5 days. Seedling growth measurements were performed at 10 days from the time of germination. To assess root length, sterilized seeds were vernalized for 2 days at 4°C and then grown for 10 days on vertically oriented MS agar plates supplemented with different concentrations of ABA. To assess ABA sensitivity during the post‐germination stages, seeds were vernalized for 2 days at 4°C and subsequently grown on MS agar medium without ABA for 2 days. Having germinated, the seeds were transferred to MS agar medium containing different concentrations of ABA and grown for a further 5 days.

To evaluate drought tolerance, pepper plants that had been infiltrated for 2 weeks and 3‐week‐old *Arabidopsis* transgenic lines were subjected to a 10‐day period during which watering was withheld, followed by 3 days during which watering was resumed. Survival rates were determined based on the number of rehydrated leaves after the period of re‐watering.

Transpiration water loss was measured using *CaADBZ1*‐silenced and control pepper plants at 2 weeks after infiltration, as well as 3‐week‐old *A. thaliana* transgenic lines. Leaves were detached and placed on Petri dishes, and the loss of fresh weight was recorded at 1‐h intervals.

To record leaf temperatures, 2‐week‐old *CaADBZ1*‐silenced and control pepper plants and 3‐week‐old *A. thaliana* plants were sprayed with 100 μM ABA, and temperatures were measured after 2 h ABA treatment using a T420 thermal imaging camera, with analysis being performed using FLIR Tools+ ver. 5.2 software.

Stomatal apertures were measured using the rosette leaves of 3‐week‐old *A. thaliana* plants and the first and second leaves of 2‐week‐old *CaADBZ1*‐silenced pepper plants. Leaf peels obtained from these leaves were floated on the stomatal opening buffer (pH 6.15, 50 mM KCl, 10 mM MES‐KOH, and 10 mM CaCl_2_) for 3 h, followed by incubation with different concentrations of ABA for an additional 3 h. Stomata were observed using a microscope, from among which we randomly selected 100 stomata for measurements of widths and lengths using ImageJ software.

### 2.7 Transactivation assay

To confirm the transactivation effect of CaADBZ1 in yeast, the full‐length or truncated coding sequence of *CaADBZ1* was cloned into the pGBKT7 vector, which contains a GAL4 DNA‐binding domain. Each construct was transformed into a yeast strain (Y2H Gold) through small‐scale yeast transformation procedures (Clontech). To select transformed yeast cells (AH109), yeast was cultured on selection media [SC–adenine–histidine–leucine–tryptophan and SC–adenine–histidine–leucine–tryptophan containing 2 mM 3‐amino‐1,2,4‐aminotriazole (3‐AT)].

For a dual‐luciferase reporter assay, the coding sequence of *CaADBZ1* was cloned into the 35S promoter‐driven pGWB2 vector as an effector and the empty pGWB2 vector as a control. The 2.94‐ and 2.83‐kb upstream sequences of the *CaOSR1* and *CaRAB18* genes are in the pGWB35 vector, which activates the Firefly luciferase (FLUC) gene used as a reporter. The *Renilla luciferase* gene was used as an internal control. Each combination of vectors was infiltrated into *N. benthamina* leaves followed by incubation for 72 h. For preparing the ABA‐treated leaf sample, a half leaf was used by the control, and the other half was sprayed with 100 μM ABA for 6 h. One side of the leaves was detached and dried for 6 hours to make drought‐treated leaf samples. The measurement of Firefly/*Renilla* (REN) luciferase activities was conducted using the Dual Luciferase™ Reporter Assay System (Promega).

## 3 RESULTS

### 3.1 Isolation and sequence analysis of CaADBZ1

**FIGURE 1:**
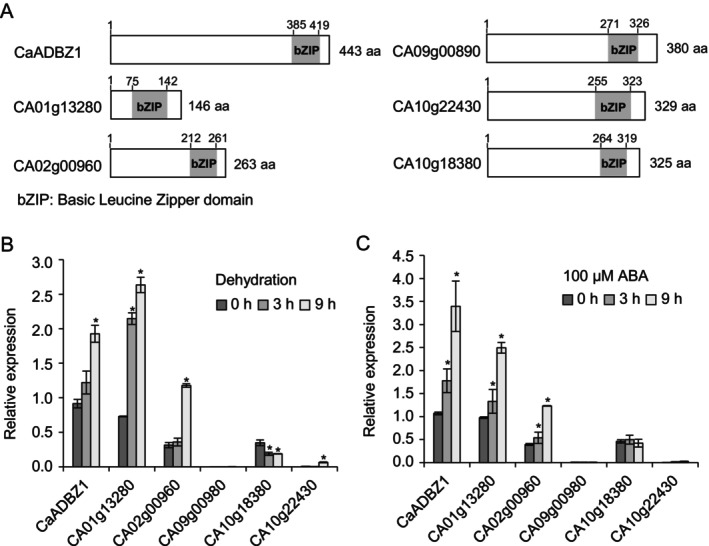
**Characterization of a pepper bZIP transcription factor**. (A) Domain organization of pepper bZIP transcription factor proteins. The amino acid sequences were obtained from SGN (http://solgenomics.net/) and domain sequences were predicted using SMART (Simple Modular Architecture Research Tool; http://smart.embl‐heidelberg.de/). (B and C) Expression levels of bZIP transcription factors in plants subjected to abiotic stresses. Pepper plants were sprayed with 100 μM solution of abscisic acid (ABA) (B). For dehydration stress, the first and second leaves were detached and dried for the indicated time (C). Expression levels were validated by qRT‐PCR analyses using cDNA derived from the first and second leaves. The pepper PP2A (*CaPP2A*) gene was used as an internal control. The expression level of *CaADBZ1* at 0 h was set to 1.0. Data are presented as the means ± standard error of three independent experiments. Asterisks indicate significant differences between untreated and treated pepper leaves (Student's *t*‐test; *p < 0.05).

In the pepper genome, 60 bZIP transcription factors have been identified, and their expressions are found to be differentially responsive to various abiotic stresses and phytohormones (Gai et al., 2020). However, the functional characterization of these transcription factors remains largely unexplored. To address this, we focused on pepper bZIP transcription factors, particularly those belonging to group A, based on the functional involvement of these members in response to various abiotic stresses (Fujita et al., 2011; Joo et al., 2021). Our aim was to isolate pepper bZIP genes involved in dehydration and ABA signalling. Through BLASTP searching with the amino acid sequences of *A. thaliana* group A bZIPs, we identified an additional bZIP gene (accession number XP_016556594) that shares sequence homology with CA09g00980 (66.1% identity/ 66.7% similarity), and named CaADBZ1 *Capsicum annuum* ABA and Drought‐Induced bZIP transcription factor 1. CaADBZ1 contains several highly conserved sequences, including the bZIP domain in the ABF2 and ABI5 genes from *Solanum lycocopersicum* and Arabidopsis thaliana (Figure S1A). These genes are bZIP transcription factors which positively modulate stress response and ABA signalling (Kim et al., 2004; Skubacz et al., 2016), so we assumed that CaADBZ1 may play a role in stress tolerance and ABA response. We also found that 28 different genera showed more than 50% identity with CaADBZ1 (Figure S1B). As shown in Figure 1A, six members of group A pepper bZIP, except CaATBZ1 (Joo et al., 2019), are characterized by the presence of a bZIP (basic‐leucine zipper) domain. To examine the response of these bZIP genes to dehydration and exogenous ABA, we treated pepper plants at the six‐leaf stage with dehydration stress and ABA. The first and second leaves were harvested at the indicated time points after treatments for RNA isolation (Figure 1B and C). qRT‐PCR analysis revealed that CaADBZ1, CA01g13280, and CA02g00960 were significantly induced by dehydration (Figure 1B) and exogenous ABA (Figure 1C). Based on its high level of induction, CaADBZ1 was selected for further study.

### 3.2 Molecular characterization of CaADBZ1

**FIGURE 2:**
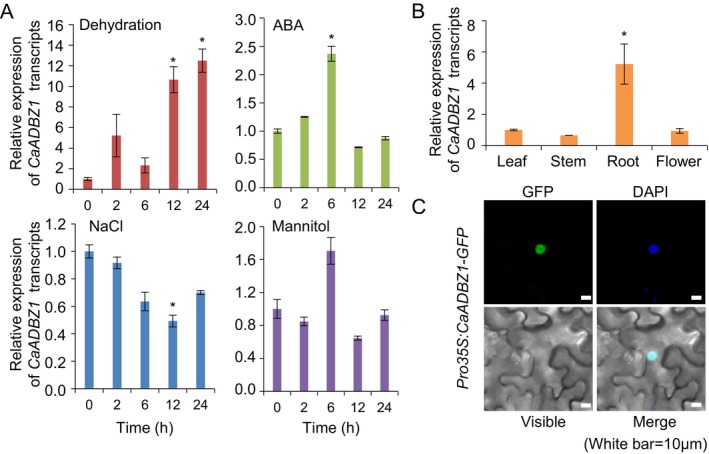
**Molecular characterization of CaADBZ1**. (A) Relative expression of *CaADBZ1* transcripts in response to different abiotic stresses. Pepper plants were treated with dehydration by detaching the first leaves, abscisic acid (ABA) (100 μM), NaCl (200 mM), or mannitol (600 mM) for the indicated time points. The pepper PP2A (*CaPP2A*) gene was used as an internal control. (B) Organ‐specific expression of CaADBZ1 in pepper plants. The relative expression of *CaADBZ1* was normalized to that of pepper PP2A (*CaPP2A*), which was used as an internal control gene. All values are presented as the means ± standard error of two independent experiments. (C) Subcellular localization of the CaADBZ1 protein. The *Pro35S:CaADBZ1‐GFP* construct was transiently expressed in tobacco leaves transformed via *Agrobacterium*‐mediated infiltration and observed under a confocal laser‐scanning microscope. White scale bar = 10 μm. The expression level of *CaADBZ1* in the leaf was set to 1.0. Data are presented as the means ± standard error of three independent experiments. Asterisks indicate significant differences between leaf and other organs (Student's *t*‐test; *p < 0.05).

Before we look over the role of CaADBZ1, we identify CaADBZ1 at the molecular level. First, we performed qRT‐PCR analyses using pepper leaves treated with dehydration, ABA, NaCl, or mannitol treatments to determine whether *CaADBZ1* transcript is responsive to environmental stimuli (Figure 2A), We found that exposure to dehydration ABA enhanced the expression of *CaADBZ1* transcripts, but salinity and osmotic treatment did not enhance the expression. Having established these responses, we subsequently examined the organ‐specific expression of *CaADBZ1* (Figure 2B), the findings of which indicated that compared with levels recorded in the leaves, stems, and flowers, *CaADBZ1* expression is more highly induced in the roots. To investigate the subcellular localization of CaADBZ1, we constructed a GFP‐tagged CaADBZ1 protein, which was transiently expressed in the leaves of *N. benthamiana* plants (Figure 2C). As revealed by the co‐localization with DAPI signals, we observed GFP signals in the nuclei of cells, thereby indicating that this protein localizes to the nucleus.

### 3.3 Transactivation activity of CaADBZ1

**FIGURE 3:**
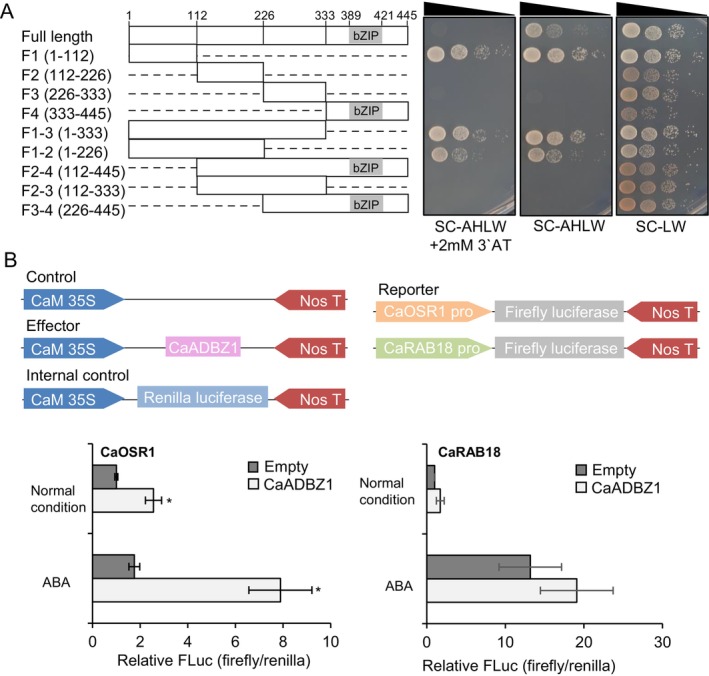
**Transient dual‐luciferase reporter assays to study the transactivation of CaADBZ1 on CaOSR1 gene**. (A) Transactivation activity of the full‐length CaADBZ1 protein and truncated forms in yeast. The yeast strain AH109 was transformed with BD‐CaADBZ1 or the respective truncated constructs (DNA‐binding domain, bait) and AD‐empty vectors (activation domain, prey), and grown on SC–leucine–tryptophan (LW) medium as a control and SC–adenine–histidine–leucine–tryptophan (AHLW) medium, with or without 2 mM 3‐amino‐1,2,4‐aminotriazole (3‐AT), for selection. Representative images were obtained after incubation at 28°C for 2 days. (B) Transient transactivation of CaADBZ1. A scheme of the firefly luciferase (FLUC) reporter vector containing the *CaOSR1* promoter region (1,500 bp) or CaRAB18 promoter region (3,519 bp) the effector vectors expressing *CaADBZ1* under the control of the 35S promoter (up). The relative FLUC/*Renilla* (REN) fluorescence ratio was determined using a reporter system (down). For exogenous ABA treatment, 100 μM ABA was sprayed for 6 h after infiltration 3 days. The value for empty vector control under normal conditions was set to 1. Asterisks indicate significant differences compared with the respective control (Student's *t*‐test; *p < 0.05).

To determine whether CaADBZ1 possesses transactivation activity and to identify which region of the CaADBZ1 sequence confers this activity, we employed a yeast hybrid assay system. A series of deletion constructs of CaADBZ1 were fused to the GAL4 DNA‐binding domain, as shown in Figure 3A. Yeast cells expressing the full‐length CaADBZ1 CDS (pGBKT7‐CaADBZ1) exhibited weak growth on the selection medium (Figure 3A), suggesting some transactivation activity. Further analysis revealed that this activity was primarily associated with the F1 fragment of CaADBZ1 (amino acids 1–112) (Figure 3A); all yeast cells harbouring constructs containing the F1 region grew well on the selection medium. These results indicate that CaADBZ1 has transactivation activity, which is largely dependent on its N‐terminal region, specifically within amino acid residues 1 to 112.

CaADBZ1 shares high sequence homology with Arabidopsis ABI5, a regulator of ABA‐responsive genes, such as *RD29B* and *RAB18* (Finkelstein & Lynch, 2000; Nakashima et al., 2006). Based on this homology, we examined whether CaADBZ1 can regulate the expression of *RAB18* and *RD29B*‐homologous gene *CaOSR1* and *CaRAB18* from pepper plants (Lim et al., 2018; Park et al., 2016) through a dual luciferase reporter assay. The upstream sequences of *CaOSR1* and *CaRAB18* were fused to the Firefly luciferase (FLUC), generating *proCaOSR1:LUC and proCaOSR1:LUC*, and these were used as a reporter. The effector *Pro35S:CaADBZ1* was transiently co‐expressed with the reporter construct (*proCaOSR1:LUC or proCaRAB18:LUC*) in *N. benthamiana* leaves. Additionally, *Pro35S:Renilla luciferase (RLUC)* was used as an internal control. As shown in Figure 3B, *CaADBZ1* expression significantly triggered the *CaOSR1* promoter‐driven *LUC* expression under both normal conditions and after ABA treatment, compared to the empty vector control. In contrast, the *CaRAB18* promoter‐driven *LUC* expression was slightly induced but not statistically significant, even after ABA treatment. Our findings suggest that CaADBZ1 has transactivation activity and may directly target the promoter of CaOSR1, not CaRAB18.

### 3.4 *CaADBZ1* ‐silenced pepper plants show reduced tolerance to dehydration stress

**FIGURE 4:**
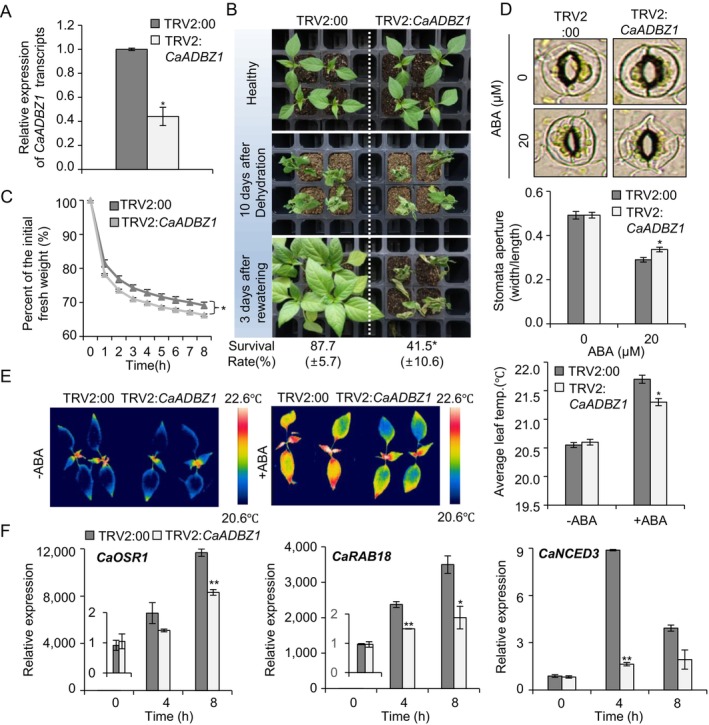
**CaADBZ1‐silenced pepper plants show reduced tolerance to dehydration stress**. (A) Quantitative RT‐PCR analysis to evaluate decreased transcript levels of CaADBZ1. Leaves of pepper plants transfected with an empty vector control (TRV2:00) or a *CaADBZ1*‐silenced construct (TRV2:*CaADBZ1*). *CaACT1* was used as an internal control gene. (B) Drought‐sensitive phenotype of TRV2:*CaADBZ1* pepper plants. Two weeks after infiltration of each line were exposed to dehydration stress by withholding watering for 10 days, followed by re‐watering for 3 days. Representative images were taken before (upper) and after (middle) drought and after 3 days of re‐watering (lower). Survival rate of TRV2:*CaADBZ1* and TRV2:00 pepper plants were measured after 3 days of re‐watering. (C) Percentage initial fresh weight of the leaves of TRV2:*CaADBZ1* and TRV2:00 plants. The leaf fresh weights of each line were measured at indicated time points after leaf detachment (*n* = 12 plants). (D) Abscisic acid (ABA)‐induced stomatal closure in TRV2:00 and TRV2:*CaADBZ1* plants. Representative images of stomatal apertures were obtained (upper) and the sizes of apertures in the plants of each line were measured (right, *n* = 100 stomata per replicate for each line). (E) Leaf surface temperatures in plants treated with 100 μM exogenous ABA. Two weeks after infiltration of each line (TRV2:00 and TRV2:*CaADBZ1*) were examined. Representative thermographic images were obtained (left) and the mean leaf temperature was measured before/after ABA treatment (right). (F) Relative transcript levels of drought‐responsive genes measured at 4 and 8 h after detaching the first leaves of TRV2:00 and TRV2:*CaADBZ1* pepper plants. The expression level of each gene in TRV2:00 at 0 h was set to 1.0. All data are presented as the means ± standard error of three independent experiments. Asterisks indicate significant differences between the TRV2:00 and TRV2:*CaADBZ1* pepper plants (Student's *t*‐test; *p < 0.05, **p < 0.01).

Having established that the expression of CaADBZ1 is promoted by dehydration treatments, we sought to investigate the dehydration‐related functions of CaADBZ1 in pepper using a VIGS system (Figure 4). To confirm the efficacy of VIGS, we initially performed a qRT‐PCR assay, the findings of which indicated a low level of *CaADBZ1* expression in *CaADBZ1*‐silenced pepper (TRV2:*CaABDZ1*) compared with that shown in the control (TRV2:00) plants (Figure 4A). Subsequently, we assessed the dehydration tolerance of these pepper plants by withholding watering for 10 days (Figure 4B). Although under both normal and after subjected dehydration, we detected no apparent phenotypic differences between TRV2:*CaABDZ1* and TRV2:00 plants (Figure 4B, upper and middle panels). Following rewatering for 3 days, TRV2:*CaABDZ1* were characterized by visibly more pronounced wilting compared to TRV2:00 (Figure 4B, lower panel); the survival of TRV2:*CaABDZ1* (41.5%) was considerably lower than that of TRV2:00 (87.7%). Given that dehydration stress resistance is closely associated with water‐holding capacity (Yamaguchi‐Shinozaki & Shinozaki, 2006), we investigated the rate of water loss over an 8‐h period by measuring the fresh weights of detached pepper leaves (Figure 4C). The fresh weights of TRV2:*CaABDZ1* leaves were lower than those of TRV2:00 leaves, indicating that TRV2:*CaABDZ1* leaves exhibit high transpiration water loss compared to those of TRV2:00. ABA‐mediated stomatal closing affects leaf water loss, which can increase dehydration resistance. (Chen et al., 2021; Kim et al., 2010; Muhammad Aslam et al., 2022). Hence, we investigated ABA sensitivity in pepper leaves by measuring stomatal apertures or leaf temperatures before and after treatment with exogenous ABA. In the absence of ABA, no significant differences were observed between TRV2:*CaABDZ1* and TRV2:00 plants. However, following exogenous ABA treatment, TRV2:*CaABDZ1* were characterized by larger stomatal pores (Figure 4D) and lower leaf temperatures (Figure 4E) than TRV2:00 plants. Considering that the induction of various stress‐responsive genes is associated with stress resistance (Khan et al., 2018), we conducted qRT PCR to analyse the expression of dehydration stress‐responsive genes in *CaADBZ1*‐silenced pepper plants. We chose *CaOSR1*, *CaRAB18*, and *CaNCED3*, which are well‐known dehydration‐induced marker genes in pepper plants (Bae et al., 2023; Baek et al., 2023). At the starting point, there are no relevant differences between TRV2:*CaABDZ1* and TRV2:00 plants. However, under dehydration conditions, the expressions of these marker genes were greatly reduced in TRV2:*CaABDZ1* than in TRV2:00 plants. These results indicate that CaADBZ1 plays a positive role in the dehydration tolerance of pepper plants by modulating ABA‐mediated stomatal closing and dehydration‐related gene expression.

### 3.5 *CaADBZ1* ‐overexpressing plants show enhanced sensitivity to ABA during seed germination and seedling growth

**FIGURE 5:**
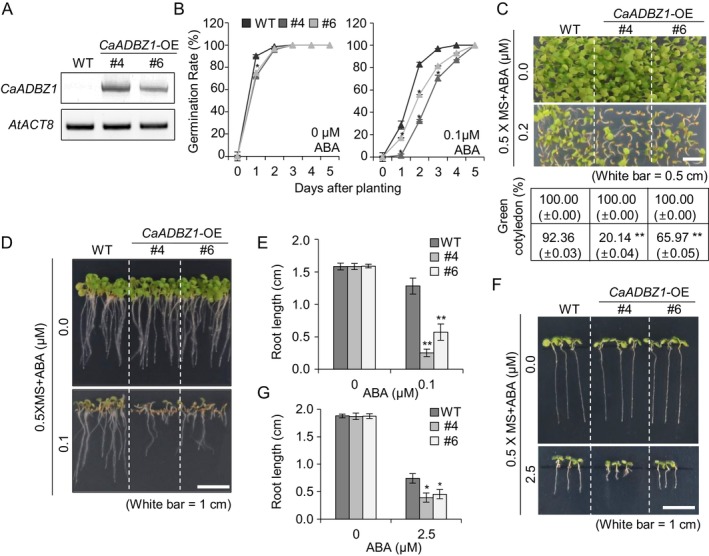
**CaADBZ1‐overexpressing Arabidopsis plants show enhanced sensitivity to abscisic acid (ABA)**. (A) Semi‐quantitative reverse‐transcription polymerase chain reaction analysis of CaADBZ1 expression in wild‐type (WT) and *CaADBZ1*‐OE transgenic lines. *Actin8* was used as an internal control gene. (B) Germination rate of wild‐type and transgenic lines in response to ABA (0.0 or 0.1 μM). The percentage of seeds showing radicle emergence was calculated. To evaluate germination rate, 72 seeds per each line were used. (C) Percentage green cotyledons of wild‐type and transgenic lines in response to ABA (0.0 or 0.2 μM). Representative images were obtained (upper) and the percentage of seedlings in each line with green cotyledons was calculated (lower) 10 days after plating. Scale bar = 0.5 cm. (D and E) Root lengths of wild‐type and transgenic lines in response to ABA (0.0 or 0.1 μM). Representative images were obtained (D) and the root lengths of each plant were measured (E) 10 days after plating. Scale bar = 1 cm. (F and G) Post‐germinative growth of wild‐type and *CaADBZ1*‐OE transgenic lines. Plants were grown on 0.5× MS medium for 2 days and following germination were transferred to 0.5× MS medium containing 0.0 or 2.5 μM ABA. Representative photographs were obtained (F) and the root lengths of each plant were measured (G) after 5 days. Scale bar = 1 cm. All values are presented as the means ± standard error of three independent experiments. Asterisks indicate significant differences between the wild‐type and *CaADBZ1*‐OE plants (Student's *t*‐test; *p < 0.05, **p < 0.01).

**FIGURE 6:**
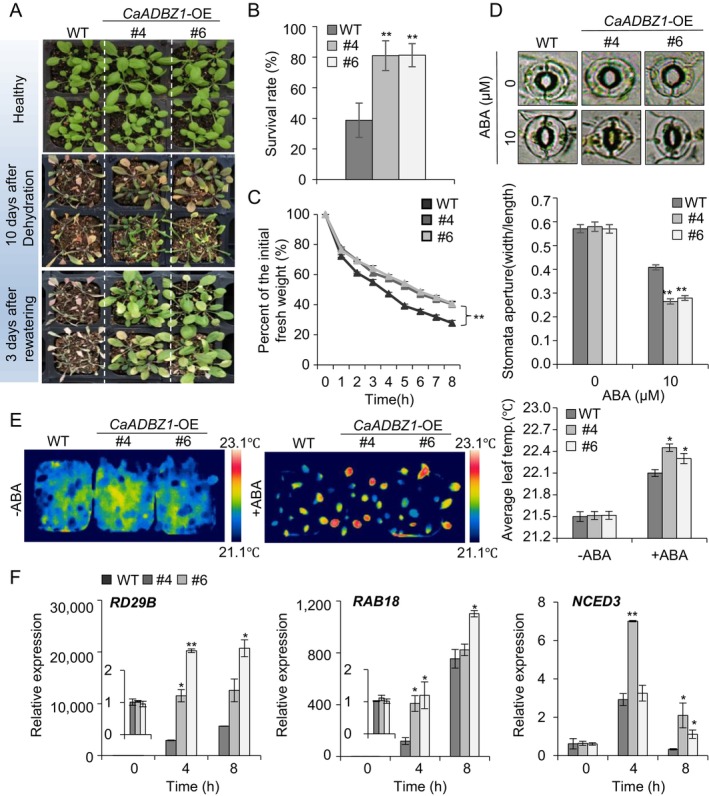
**CaADBZ1‐overexpressing (OE) Arabidopsis plants show enhanced tolerance to dehydration stress**. (A and B) Drought‐tolerant phenotype of *CaADBZ1*‐OE *Arabidopsi*s plants. Three‐week‐old wild‐type (WT) and *CaADBZ1*‐OE transgenic plants of each line were subjected to dehydration stress by withholding watering for 10 days, followed by re‐watering for 3 days. (A) Representative images were taken before (upper) and after (middle) drought and after 3 days of re‐watering (lower). (B) Survival rates of wild‐type and *CaADBZ1*‐OE transgenic plants after 3 days of re‐watering. (C) Percentage initial fresh weight of the rosette leaves of wild‐type and *CaADBZ1*‐OE lines at different time points after detachment (n = 10 plants). (D) Abscisic acid (ABA)‐induced stomatal closure in wild‐type and transgenic plants. Representative images of stomatal apertures were obtained (top) and the stomatal apertures in the leaves of each line were measured (bottom). (E) Leaf temperatures of wild‐type and *CaADBZ1*‐OE transgenic plants following ABA treatment. Representative thermographic images were taken at 2 h after treatment with 100 μM ABA. (F) Relative transcript levels of drought‐responsive genes measured at 4 and 8 h after detaching the rosette leaves of wild type and *CaADBZ1*‐OE transgenic plants. The expression level of each gene in wild type at 0 h was set to 1.0. All values are presented as the means ± standard error of three independent experiments. Asterisks indicate significant differences between the wild‐type and *CaADBZ1*‐OE plants (Student's *t*‐test; *p < 0.05, **p < 0.01).

We further examined the biological roles of CaADBZ1 by generating *CaADBZ1‐*overexpressing (OE) *Arabidopsis* plants (Figures 5 and 6). Initially, we determined the levels of *CaADBZ1* in two independent transgenic lines (*CaADBZ1‐*OE #4 and #6) through RT‐PCR analyses (Figure 5A). These two Arabidopsis plant lines showed significantly higher expression of *CaADBZ1* compared to wild‐type (Figure 5A), and were subsequently used for phenotypic analyses. To assess ABA sensitivity during seed germination and seedling growth, seeds of *CaADBZ1‐*OE and wild‐type plants were sowed onto ½ MS media containing 0 and 0.1 μM ABA. (Figure 5). In the absence of ABA, we detected no significant differences between wild‐type and *CaADBZ1‐*OE plants. However, *CaADBZ1*‐OE seeds displayed a delayed germination rate and a lower rate of cotyledon greening compared to wild‐type seeds in the presence of 0.1 μM ABA (Figure 5B and C). This enhanced ABA sensitivity was further evident in the significantly reduced primary root growth observed in *CaADBZ1*‐OE plants compared to wild‐type plants (Figure 5D and E). To determine if this hypersensitivity was solely caused by the delay in germination, two‐day‐old seedlings of *CaADBZ1*‐OE and wild‐type grown in the absence of ABA were transferred to ½ MS media supplemented with 0 and 2.5 μM ABA. Following growth for a further 5 days, root lengths of *CaADBZ1‐*OE plants were significantly shorter than those of wild‐type plants (Figure 5F and G). These findings suggest that CaADBZ1 positively contributes to the enhanced ABA sensitivity at both germinative and post‐germinative stages.

### 3.6 *CaADBZ1‐* OE plants show enhanced tolerance to dehydration stress

Next, we investigated how overexpression of the CaADBZ1 gene affects the responses of Arabidopsis plants to dehydration stress (Figure 6). Under well‐watered conditions, we detected no significant phenotypic differences between wild‐type and *CaADBZ1‐*OE plants (Figure 6A, upper panel). As described above, dehydration stress was treated by withholding watering for 10 days and subsequently re‐watering for 3 days. We found that compared to the wild‐type plants, *CaADBZ1‐*OE plants displayed less wilting (Figure 6A, middle and lower panels), and the survival of *CaADBZ1‐*OE plants (81.0%–81.3%) was notably higher than that of the wild‐type plants (38.8%) (Figure 6B). To investigate the water retention capacity of these plants, we measured the rate of water loss from detached rosette leaves at different time points. Compared to wild‐type plants, the *CaADBZ1‐*OE plants were characterized by consistently lower rates of water loss (Figure 6C). To determine whether the reduced loss of water was attributed to stomatal regulation, we measured stomatal apertures and leaf temperatures in the presence or absence of ABA (Figure 6D and E). Before the application of exogenous ABA, we detected no significant differences between *CaADBZ1‐*OE and wild‐type plants. However, when exposed to exogenous ABA, the stomatal apertures of *CaADBZ1‐*OE plants were notably smaller than those of wild‐type plants (Figure 6D). Furthermore, compared to wild‐type plants, we recorded higher temperatures in the leaves of *CaADBZ1‐*OE plants after ABA treatment (Figure 6E). Consistently, Arabidopsis dehydration‐responsive genes, including *RD29B*, *RAB18*, and *NCED3, were* more highly induced in *CaADBZ1‐OE* plants than in wild‐type plants (Figure 6F). These findings indicate that CaADBZ1 enhances dehydration tolerance by increasing ABA‐mediated stomatal closure and inducing the expression of dehydration‐responsive genes.

## 4 DISCUSSION

As a strategy for adapting to unfavourable environments, plants can modulate the expression of a range of abiotic stress‐related genes. In this regard, the plant hormone ABA has been established to play diverse important roles in the defence mechanisms induced in response to abiotic stresses, such as drought (Finkelstein, 2013; Ng et al., 2014). Transcription factors (TFs) are crucial in regulating drought‐responsive genes by interacting with their promoter regions (Singh & Laxmi, 2015; Sornaraj et al., 2016). Among these, bZIP family TFs are highly conserved and prevalent in plants. Previous studies have demonstrated that bZIP TFs contribute to abiotic stress responses by enhancing ABA sensitivity (Duan et al., 2022; Finkelstein & Rock, 2002; Nijhawan et al., 2008). In particular, ABI5 has been shown to modulate ABA signalling in seeds, whereas ABFs play a similar modulatory role in vegetative tissues (Fujita et al., 2005; Furihata et al., 2006; Lopez‐Molina & Chua, 2000). However, the functions of a majority of bZIP TFs remain poorly understood.

In this study, we isolated six pepper group A bZIP transcription factors and selected CaADBZ1 for further analysis due to its strong induction in response to both exogenous ABA and dehydration treatments (Figure 1). Our qRT‐PCR analyses revealed a significant increase in CaADBZ1 transcript level upon exposure to dehydration and ABA but not in response to NaCl and mannitol treatments (Figure 2A). Group A bZIP TFs, including ABI5 and ABFs, have three conserved regions (C1, C2, and C3) at the N‐terminus and one conserved region (C4) at the C‐terminus, in addition to the bZIP domain (Bensmihen et al., 2002; Uno et al., 2000; Yoshida et al., 2015). Among them, the C1 region is shown to play an important role in transactivation activity (Nakamura et al., 2001; Yue et al., 2019). Consistently, CaADBZ1 also contains a highly conserved C1 region (red box as shown below in Figure S1B) at the N‐terminus, and our results confirmed that the F1 fragment containing the C1 region was needed for the transactivation activity of CaADBZ1. We conducted a dual luciferase reporter assay to assess the transactivation activity of CaADBZ1 in planta. The expression of two dehydration/ABA‐responsive genes, *RD29B* and *RAB18*, is known to be regulated by ABI5 (Brocard et al., 2002; Lång & Palva, 1992; Park et al., 2016). CaADBZ1 sequence shares homology with Arabidopsis ABI5 (Figure S1B), and we confirmed that CaADBZ1 enhanced *LUC* expression driven by the promoter of *CaOSR1*, a homolog of *RD29B*, as a reporter under ABA treatment compared to the control (Figure 3B). Under normal conditions, LUC expression increased by 2.75‐fold, while under ABA treatment, it increased by 4.48‐fold. However, no significant effect of *CaADBZ1* on *CaRAB18* promoter‐driven LUC expression was observed, suggesting that CaADBZ1 directly modulates *CaOSR1* gene expression in ABA signalling.

The induction of drought‐responsive gene by CaADBZ1 positively contributes to drought tolerance. *CaADBZ1*‐silenced peppers exhibited reduced drought tolerance, while CaADBZ1‐OE Arabidopsis plants showed enhanced drought tolerance (Figures 4B and 6A). These plants also displayed differences in ABA sensitivity, as indicated by variations in leaf fresh weight due to differential transpiration water loss. Since ABA plays an essential role in governing stomatal opening and closure, which in turn regulates plant water potential (Fang & Xiong, 2015), we found that *CaADBZ1*‐silenced pepper and *CaADBZ1*–OE *Arabidopsis* were accordingly observed to have larger and smaller stomatal pores, respectively (Figures 4D and 6D), accounting for the observed differences in transpiration water loss and drought tolerances. Moreover, *CaADBZ1*‐OE plants were characterized by heightened sensitivity to ABA during germination and seedling stages.

Numerous ABA‐responsive genes are modulated by various transcription factors expression in ABA signalling (Hussain et al., 2021). For example, WRKY transcription factors can bind other transcription factors and modulate their expression in the early stage of ABA signalling (Rushton et al., 2012). One of the WRKY transcription factors, AtWRKY40, can bind the ABI5 promoter region and repress ABI5 (Shang et al., 2010). Similarly, APETALA2/ETHYLENE RESPONSIVE FACTOR (AP2/ERF) family members, such as RAV1, modulate drought tolerance by repressing ABI3, ABI4, and ABI5, thus reducing ABA sensitivity (Feng et al., 2014). CaADBZ1 has a well‐conserved bZIP domain (Supplementary Figure 1A) and has transactivation activity (Figure 3). We further examined its role in regulating dehydration‐responsive genes in pepper plants, including *CaOSR1*, *CaRAB18*, and *CaNCED3*. *RD29B*, a homolog of *CaOSR1*, and *RAB18* are LEA genes, both modulated by ABF transcription factors and are involved in seed maturation and dehydration resistance (Bojórquez‐Velázquez et al., 2019; Sun et al., 2021). *NCED3* encodes a key enzyme in ABA synthesis (Qin & Zeevaart, 1999; Thompson et al., 2000). CaADBZ1 was shown to affect the expression of *CaOSR1*, *CaRAB18*, and *CaNCED3* genes. These genes exhibited more than 3.46‐fold increased expression in *CaADBZ1*‐OE plants, while expression was reduced by over 1.75‐fold in *CaADBZ1*‐silenced pepper (Figures 4F and 6F). Despite the lack of direct luciferase activity for CaRAB18, its expression level was altered, suggesting an indirect regulation by CaADBZ1.

In conclusion, we identified and characterized CaADBZ1, a pepper bZIP transcription factor that enhances drought tolerance by modulating ABA sensitivity and regulating the expression of ABA‐responsive genes, particularly *CaOSR1*. Further studies are needed to identify CaADBZ1 interactors and elucidate how these interactions influence the dehydration response in pepper plants. These studies will make an important contribution to our current understanding of the biological function of bZIP transcription factors in ABA signalling and plant defence mechanisms in dehydration.

## AUTHOR CONTRIBUTIONS

J.C., C.W.L., and S.C.L. conceived the research; J.C. and C.W.L. performed the experiments; J.C. and C.W.L. analyzed the data; and J.C. and S.C.L. wrote the manuscript. All authors have reviewed and approved the final manuscript.

## FUNDING INFORMATION

This work was supported by a grant from the Agriculture & Technology Development (Project No. RS‐2024‐00322140) and a National Research Foundation of Korea (NRF) grant funded by the Korean Government (No. RS‐2024‐00343006), Republic of Korea.

## CONFLICT OF INTEREST STATEMENT

The authors have no conflicts of interest to declare.

## Supporting information


**Supplementary Table 1.** Sequences of the primers used in this study.
**Supplementary Figure 1. Analysis of the protein sequences of CaADBZ1**. (A) Alignment of the sequences of the CaADBZ1 protein and homologous proteins (Capsicum *chinense*; accession no. PHU06257.1; *Solanum lycopersicum*; accession no. XP_010325918.1; *Solanum tuberosum*; accession no. KAH0632400.1; *Nicotiana attenuata*; accession no. XP_019250839.1; *Nicotiana tabacum*; accession no. XP_016434321.1; Arabidopsis *thaliana*: *accession* no. AT2G36270) using MEGA software (version 10.1.8). Identical and similar residues in sequences are indicated in black. The red‐lined boxes indicate the Basic leucine zipper domain. The boxes which lined various colors (orange, green, sky blue and purple) indicate regions which were well‐conserved in group A b ZIP transcription factors. (B) Phylogenetic tree analysis of the CaADBZ1 protein. A BLAST search was conducted using the amino acid sequence of CaADBZ1 and database sequences showing the highest similarity were selected. MEGA software (version 10.1.8) was used to conduct multiple sequence alignment of the selected amino acid sequences and for the construction of the phylogenetic tree. Bootstrap values are indicated at each branch point, which were calculated from 1,000 bootstrap replications. Genetic distance is indicated by the scale bar.

## Data Availability

All data are available in the manuscript or Supplementary files. The raw data that support the findings of this study are available from the corresponding author upon reasonable request.
