# Supplementary material for: Role of pepper bZIP transcription factor CaADBZ1 in abscisic acid signalling and drought stress response
Source: Physiol Plant. 2025 Mar 19;177(2):e70159. doi: 10.1111/ppl.70159 (PMC11920937; doi:10.1111/ppl.70159)
Supplement: Supplementary file 1 — Supplementary Table 1. Sequences of the primers used in this study. Supplementary Figure 1. Analysis of the protein sequences of CaADBZ1. (A) Alignment of the sequences of the CaADBZ1 protein and homologous proteins (Capsicum chinense; accession no. PHU06257.1; Solanum lycopersicum; accession no. XP_010325918.1; Solanum tuberosum; accession no. KAH0632400.1; Nicotiana attenuata; accession no. XP_019250839.1; Nicotiana tabacum; accession no. XP_016434321.1; Arabidopsis thaliana: accession no. AT2G36270) using MEGA software (version 10.1.8). Identical and similar residues in sequences are indicated in black. The red‐lined boxes indicate the Basic leucine zipper domain. The boxes which lined various colors (orange, green, sky blue and purple) indicate regions which were well‐conserved in group A b ZIP transcription factors. (B) Phylogenetic tree analysis of the CaADBZ1 protein. A BLAST search was conducted using the amino acid sequence of CaADBZ1 and database sequences showing the highest similarity were selected. MEGA software (version 10.1.8) was used to conduct multiple sequence alignment of the selected amino acid sequences and for the construction of the phylogenetic tree. Bootstrap values are indicated at each branch point, which were calculated from 1,000 bootstrap replications. Genetic distance is indicated by the scale bar. [file PPL-177-e70159-s001.pdf]

Supplementary Table 1. Sequences of the primers used in this study

Primer name		Primer sequence (5`-3`)
**For cloning**		
CaADBZ1-CDS	Forward	ATGGGAGTAACAGAATCAGAGATGGT
	Reverse	TCAAGGGCAACTAAAGCTCCTC
CaADBZ1-w/o stop codon	Forward	GGTCGAATTCGCCCTTAGGGCAACTAAAGCTC
	Reverse	GAGCTTTAGTTGCCCTAAGGGCGAATTCGACC
CaADBZ1-VIGS	Forward	TCTAGAATGGGAGTAACAGAATCAGAG
	Reverse	CTCGAGAGGCTGAGCATGAGAAT
**For Transactivation activity of CaADBZ1**		
F1	Forward	ATGGGAGTAACAGAATCAGAGATGGT
	Reverse	TCATGGCTTCCCCGGCACTTA
F2	Forward	ATGCCAGCGCGGCGCCACATT
	Reverse	TCAATACATCGTATATGACTGCTGCTGAG
F3	Forward	ATGCAAAACAGTACAGCTCCCACTAT
	Reverse	TCACCCGCTATCGAGTTGATTCACGCA
F4	Forward	ATGGGCCAATACGGGTTGGAA
	Reverse	TCAAGGGCAACTAAAGCTCCTC
**For RT-PCR**		
CaADBZ1	Forward	GGAGTTGGCGTTGGCATTCCT
	Reverse	GTCTAGTACGCGTTTTCGCCCG
CA01g13280	Forward	CAAAATTATGTGCCTCCGACAGG
	Reverse	CCTCTTCCAGATGTGAAACCTTGTTC
CA02g00960	Forward	TTTGGAGGCGACTTCGATGACG
	Reverse	ATAGCCACTGACTCCTCCTCAACCG
CA09g00890	Forward	CTCGAGGATTTCTTGGTTAAAGCAG
	Reverse	CTCCCACCGTAAACTGGCGTTG
CA10g18380	Forward	GACTGTATGGACTGCTGAGGCTAGTCA
	Reverse	CCTAAACTTTTCTTCCCCGGAT
CA10g22430	Forward	TGTACGGACTATTGAGGCTAATCAAGA
	Reverse	TTAACCCCTGATACTAAACCCGAAT
CaNCED3	Forward	TTAAGGATCTTAAGCGTGGTTATGT
(CA08g03620)	Reverse	AGATTAGTTCAAGAACGTGAATTGG
CaOSR1	Forward	ATGGAGGCACAACTGCACCGTC
(CA03g17780)	Reverse	GGCCCACCATGAACTTCTGCAC
CaRAB18	Forward	ATGTCGCACTACGAGAACCAATATAG
(CA02g22060)	Reverse	ATCATCCTCAGAGCTGCTGGAGC
CaACT1	Forward	GACGTGACCTAACTGATAACCTGAT
	Reverse	CTCTCAGCACCAATGGTAATAACTT
AtACT8	Forward	CAACTATGTTCTCAGGTATTGCAGA
	Reverse	GTCATGGAAACGATGTCTCTTTAGT
RD29B	Forward	GTTGAAGAGTCTCCACAATCACTTG
	Reverse	ATACAAATCCCCAAACTGAATAACA
RAB18	Forward	GGAAGAAGGGAATAACACAAAAGAT
	Reverse	GCGTTACAAACCCTCATTATTTTTA
NCED3	Forward	ACATGGAAATCGGAGTTACAGAT
	Reverse	AGAAACAACAAACAAGAAACAGAGC

A

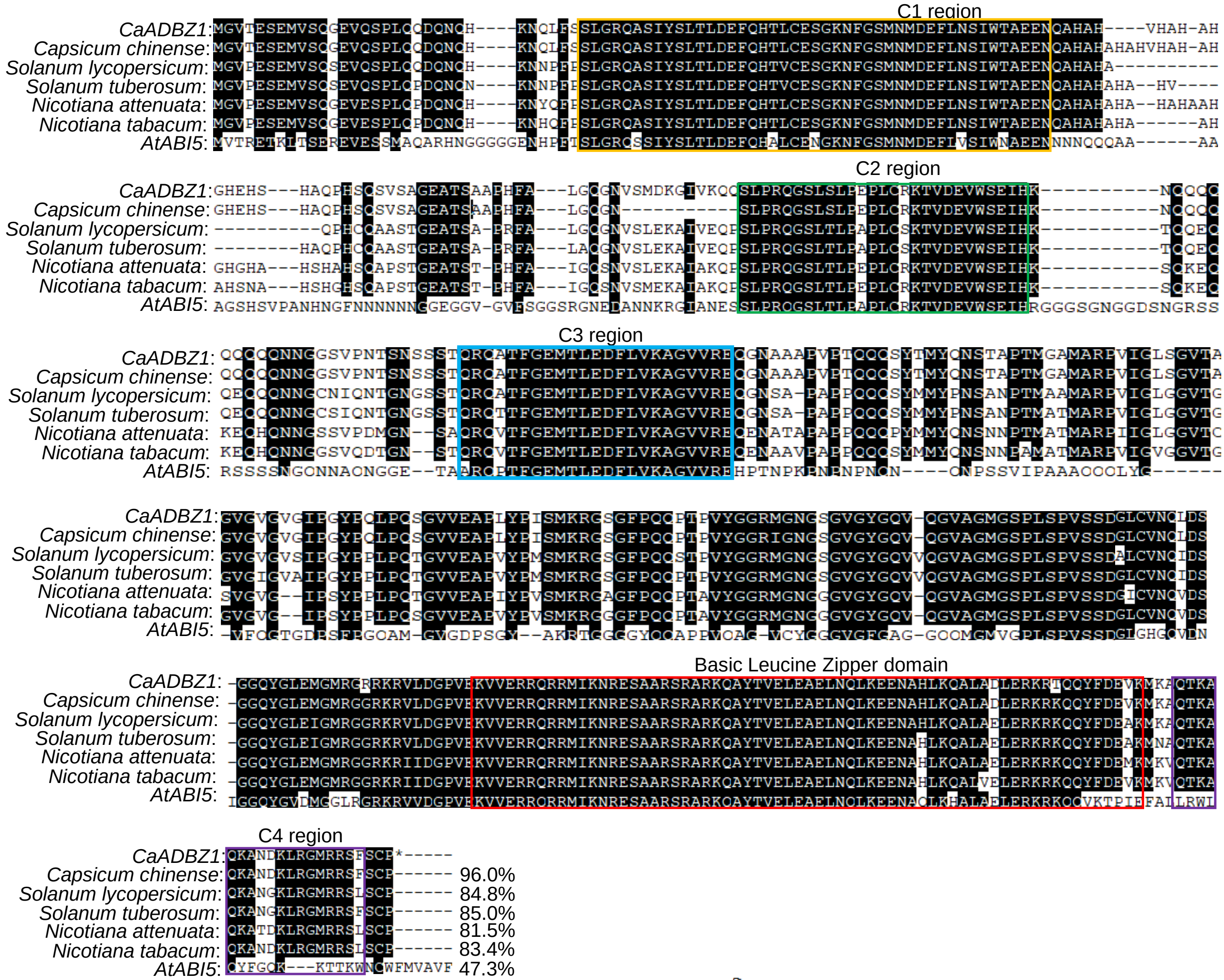

B

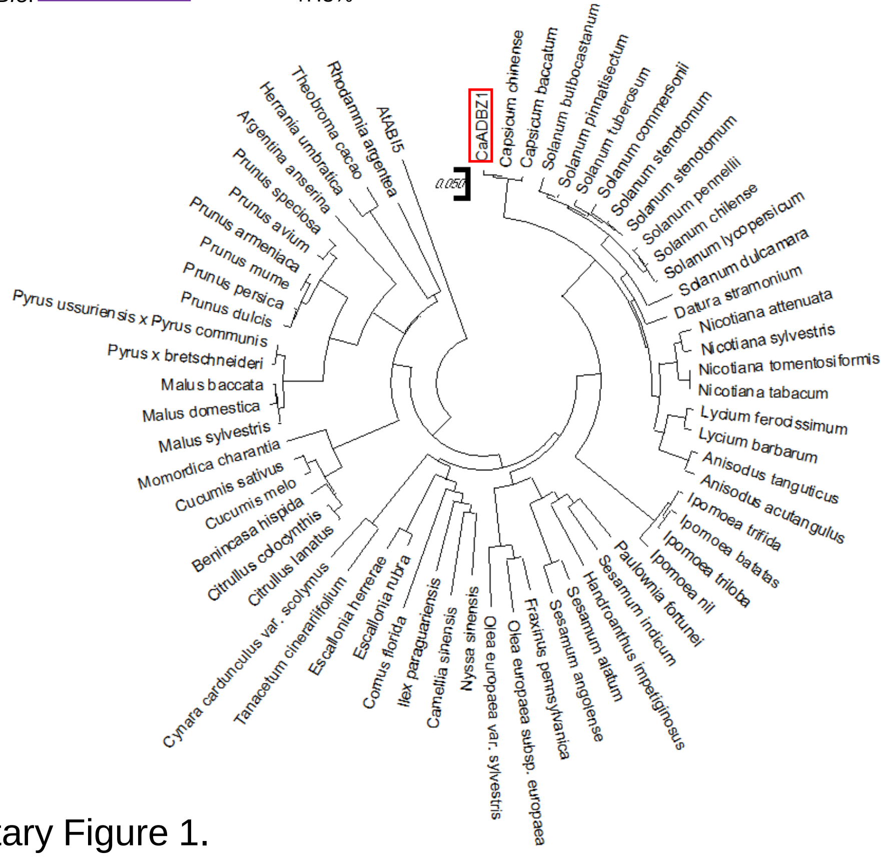

Supplementary Figure 1.
